# Measurement of the critical DNA lesions produced by antibody-directed enzyme prodrug therapy (ADEPT) in vitro, in vivo and in clinical material

**DOI:** 10.1054/bjoc.2001.1843

**Published:** 2001-06

**Authors:** S D Webley, R J Francis, R B Pedley, S K Sharma, R H J Begent, J A Hartley, D Hochhauser

**Affiliations:** Department of Oncology, CRC Drug–DNA Interactions Research Group and CRC Targeting and Imaging Group, Royal Free and University College School of Medicine,University College London for the Phase I and II Clinical Trials Committee of the Cancer Researchampaign, UK

**Keywords:** colorectal cancer, antibody, ADEPT, comet assay, interstrand crosslink

## Abstract

An antibody-directed enzyme prodrug therapy (ADEPT) system against CEA-positive tumours is currently in phase I clinical trials. It consists of a prodrug, 4-[N,N-bis(2-iodoethyl) amino] phenoxycarbonyl L -glutamic acid (ZD2767P) and a conjugate of the F(ab')_2_ anti-CEA antibody A5B7 and the bacterial enzyme carboxypeptidase G2 (CPG2). ZD2767P is converted by antibody-targeted CPG2 into an active bifunctional alkylating drug (ZD2767) at the tumour site. The IC _50_ value of the prodrug against the human colorectal tumour LS174T cell line was 55 ± 9 μM following a 1 h exposure. In contrast, co-incubation of ZD2767P with CPG2 resulted in 229-fold increase in activity. Using a modified comet assay, DNA interstrand cross links (ISC) were detected within 1 h of ZD2767P + CPG2 treatment and were repaired by 24 h. A clear dose–response was seen between the level of ISC, growth inhibition and ZD2767 concentration. Administration of a therapeutic dose of ZD2767P 72 h after the F(ab′)_2_ A5B7 conjugate to mice bearing LS147T xenografts resulted in extensive ISC in the tumour after 1 h; repair was seen at 24 h. Tumour biopsies and peripheral lymphocytes were studied in 5 patients on the ADEPT phase I clinical trial. In 4 patients no ISC were detected. These patients also demonstrated poor localization of conjugate and no tumour response was seen. However a significant level of ISC was detected in one tumour biopsy, which also showed evidence of conjugate localization and clinical response. These studies demonstrate the application of the comet assay in the measurement of ISC in vitro and in clinical material and confirm that activation of ZD2767P results in the formation of DNA crosslinks. © 2001 Cancer Research Campaign http://www.bjcancer.com


British Journal of Cancer (2001) 84(12), 1671–1676
© 2001 Cancer Research Campaign
doi: 10.1054/ bjoc.2001.1843, available online at http://www.idealibrary.com on IDEAL®

### Measurement of the critical DNA lesions produced by antibody-directed enzyme prodrug therapy (ADEPT) in vitro, in vivo and in clinical material

**SD Webley, RJ Francis, RB Pedley, SK Sharma, RHJ Begent, JA Hartley and D Hochhauser**

CRC Drug–DNA Interactions Research Group and CRC Targeting and Imaging Group, Department of Oncology, Royal Free and University College School of Medicine, University College London for the Phase I and II Clinical Trials Committee of the Cancer Research Campaign, UK

**Summary** An antibody-directed enzyme prodrug therapy (ADEPT) system against CEA-positive tumours is currently in phase I clinical trials. It consists of a prodrug, 4-[N,N-bis(2-iodoethyl) amino] phenoxycarbonyl L-glutamic acid (ZD2767P) and a conjugate of the F(ab’)$_2$ anti-CEA antibody A5B7 and the bacterial enzyme carboxypeptidase G2 (CPG2). ZD2767P is converted by antibody-targeted CPG2 into an active bifunctional alkylating drug (ZD2767) at the tumour site. The $IC_{50}$ value of the prodrug against the human colorectal tumour LS174T cell line was 55 ± 9 μM following a 1 h exposure. In contrast, co-incubation of ZD2767P with CPG2 resulted in 229-fold increase in activity. Using a modified comet assay, DNA interstrand cross links (ISC) were detected within 1 h of ZD2767P + CPG2 treatment and were repaired by 24 h. A clear dose–response was seen between the level of ISC, growth inhibition and ZD2767 concentration. Administration of a therapeutic dose of ZD2767P 72 h after the F(ab′)$_2$ A5B7 conjugate to mice bearing LS147T xenografts resulted in extensive ISC in the tumour after 1 h; repair was seen at 24 h. Tumour biopsies and peripheral lymphocytes were studied in 5 patients on the ADEPT phase I clinical trial. In 4 patients no ISC were detected. These patients also demonstrated poor localization of conjugate and no tumour response was seen. However a significant level of ISC was detected in one tumour biopsy, which also showed evidence of conjugate localization and clinical response. These studies demonstrate the application of the comet assay in the measurement of ISC in vitro and in clinical material and confirm that activation of ZD2767P results in the formation of DNA crosslinks. © 2001 Cancer Research Campaign http://www.bjcancer.com

**Keywords**: colorectal cancer; antibody; ADEPT; comet assay; interstrand crosslink

ADEPT represents a potentially valuable means of effectively destroying tumours without causing systemic toxicities (Sherwood, 1996; Bagshawe et al, 1998). The key element in this strategy is tumour specificity; tumours are selectively targeted through the antigens they express. ADEPT is a 2-step process which ultimately leads to the production of high concentrations of active drug selectively at the tumour site. The first step involves the administration of an antibody-enzyme conjugate which is allowed to localize at the tumour site and clear from normal tissue. A relatively nontoxic prodrug is then administered and is converted by the enzyme to a cytotoxic drug by prelocalized enzyme. Both bacterial and mammalian enzymes have been evaluated as potential candidates for ADEPT, but so far, the bacterial enzyme carboxypeptidase has been utilized in ADEPT clinical development due to its superior activity in vitro and in animal studies. The enzyme has no known mammalian equivalent and catalyses the hydrolytic cleavage of glutamyl residues. Earlier work has previously demonstrated that a conjugate of CPG2 linked to the anti-CEA antibody, A5B7 in combination with benzoic acid mustard glutamate prodrugs resulted in significant antitumour activity (Blakey et al, 1993). Responses were reported following a pilot clinical study of patients with colorectal cancer using CPG2-F(ab′)2 A5B7 conjugate to activate the benzoic acid mustard prodrug prodrug 4-[2-chloroethyl-(2mesyloxyethyl)amino] benzoyl-L-glutamic acid (CMDA) (Napier et al, 2000).

*Received 27 November 2000*
*Revised 19 March 2001*
*Accepted 20 March 2001*

*Correspondence to:* D Hochhauser

Animal models and previous clinical studies have demonstrated that ADEPT can be used to selectively deliver an antibody-enzyme conjugate to a tumour and produce significant regression (Springer et al, 1991a; Eccles et al, 1994). These effects have not, however, been correlated directly with production of the prodrug. The ADEPT clinical trial sponsored by the CRC and AstraZeneca commenced at the Royal Free Hospital in November 1997. The formulation includes the bisiodophenol mustard ZD2767 which has replaced the previously used CMDA. ZD2767 has the advantages of increased potency and decreased half-life (Springer et al, 1995).

The active drug produced from ZD2767P is a nitrogen mustard. Nitrogen mustards have been extensively studied as chemotherapeutic agents. Their primary mode of action is through alkylation of DNA and, in particular, the production of DNA interstrand crosslinks (ISC). The level of ISC has been shown to correlate with cytotoxicity in in vitro studies (Sunters et al, 1992). Although the quantitation of DNA ISC is relatively easy in vitro with techniques such as alkaline filter elution, these methods are generally not applicable to clinical studies. The single cell gel electrophoresis (comet) assay has become established as a sensitive method to analyse DNA strand breakage in vitro and in vivo (Fairbairn et al, 1995). Recently we have optimized a modification of this method to allow the detection of ISC following drug exposure (Hartley et al, 1999). Cells are irradiated immediately prior to analysis to deliver a fixed level of random strand breakage and crosslinks are quantitated as the decrease in the comet tail moment compared to unirradiated controls. The method is more sensitive than alkaline elution and can be used to assess clinical samples. It also allows evaluation of heterogeneity in clinical specimens. For example the formation and repair of ISC has been studied in the lymphocytes of patients treated with ifosfamide (Hartley et al, 1999).

Nitrogen mustards are good candidates for use in ADEPT since they kill both quiescent and proliferating cells. Despite their suitability for ADEPT, nitrogen mustards prodrugs, when activated, tend to have a long chemical half life which results in non-tumour tissue toxicity (Springer et al, 1991b). The bisiodophenol mustard ZD2767 represents an improved prodrug component of ADEPT compared to the original benzoic acid mustard CMDA and the 4-[N,N-bis(2-chloroethyl)amino]-phenol prodrugs. When cleaved by CPG2, ZD2767P produces a highly reactive nitrogen mustard which has a short half life of 2 minutes in plasma (Blakey et al, 1996). Combination of CPG2-anti-CEA congugate and ZD2767P has been reported to result in improved antitumour activity in a colorectal tumour xenograft model and is currently in phase I clinical trial.

In the current study we sought to determine the major DNA lesions produced by the ZD2767P ADEPT system and to establish the kinetics of DNA damage and repair following treatment with this compound. In so doing, the present study has highlighted the value of the comet assay for measuring DNA lesions in vivo in both animal model systems and in clinical samples.

### MATERIALS AND METHODS

### Materials

All standard laboratory chemicals used in this study were commercial products of AnalaR® grade purchased from Sigma (Poole, UK). ZD2767P bisiodophenol was synthesized as previously published (Springer et al, 1991a). The F(ab′)2 fragment of A5B7 was conjugated to the bacterial enzyme CPG2 as described previously (Melton et al, 1993).

CPG2 was produced as previously described (Sherwood et al, 1985) and kindly provided by Dr Roger Melton from the Division of Biotechnology, CAMR, Porton Down, UK.

### Cell culture

The human colorectal tumour cell line LS147T was maintained in DMEM culture medium and 10% FCS supplemented with 2 mM glutamine, at 37˚C in air containing 5% $CO_2$. The cells were routinely subcultured once a week.

### Growth inhibition studies

The sulphorhodamine B (SRB) assay (Skehan et al, 1990) was used to determine growth inhibition. The cells were seeded at 1 to $2 \times 10^3$ cells per well in a 96-well plate and left to adhere overnight prior to drug exposure. Drug exposure was for 1 h, after which time the cells were incubated at 37˚C for a further 4 days and then fixed with 50 µl trichloroacetic acid for 20 minutes. The cells were washed 5 times with tap water before incubation with 0.4% SRB solution in 1% acetic acid. After washing 5 times in 1% acetic acid, the plates were air dried overnight. The SRB stained cells were then dissolved in 100 µl 10 mM Tris and the plates read at 540 nm using a plate reader.

### Measurement of interstrand crosslinks using the comet assay

The comet assay was performed under alkaline conditions as described with modifications (Spanswick et al, 1998). Briefly, $2.4 \times 10^4$ cells were embedded in 1% agarose on a microscope slide then lysed in high salt buffer containing 2.5 M NaCl, 100 mm disodium EDTA, 10 mM Tris-HCl (pH 10.5), 1% triton X-100. After washing with $H_2O$ for 1 h, the cells were exposed to alkaline buffer containing 50 mM NaOH, 1 mM disodium EDTA (pH 12.5) for 40 min before electrophoresis at 18 V (20 min). The DNA was then stained with propidium iodide and visualized using a fluorescent microscope. The Komet image analysis system was used to measure the olive tail moment as percentage of DNA in the tail multiplied by the comet length. For ISC measurements, samples were first given a known dose of irradiation (10 Gy) before comet analysis. The presence of ISC was seen as the reduction in tail moment of the irradiated treated samples compared to the irradiated untreated control samples. ISC were therefore quantified as the percentage reduction in tail moment. Experiments in the absence and presence of proteinase K have shown that he comet assay as described measures interstrand crosslinks.

### In vivo studies

The human colon adenocarcinoma cell line LS174T was used to develop a xenograft model in the flank of female nude mice. Subsequent passaging was by subcutaneous implantation of small tumour pieces (~1 $mm^3$). The tumour is a moderately differentiated CEA-producing adenocarcinoma with small glandular acini which secretes no measurable CEA into the circulation. All mice were aged 2–3 months and weighed 20–23g at initiation of the experiments. The experiments commenced when the tumours were between 0.5–0.75 $cm^3$ at approximately 2 weeks after passaging. The F(ab′)2 fragment of A5B7 was conjugated to the bacterial enzyme CPG2 as previously described (Melton et al, 1993). 1 Unit of activity is defined as the amount of enzyme required to catalyse the hydrolysis of 1 $\mu mol\ min^{-1}\ ml^{-1}$ methotrexate in reaction mixture (Sherwood et al, 1985). Conjugate (25 U/mouse) was injected intravenously into the tail vein. CPG2 levels were monitored indirectly by a spectrophotometric assay (Sherwood et al, 1985) and once a level of 0.1 U $ml^{-1}$ had been reached (72 h later) 75 mg $kg^{-1}$ or 45 mg $kg^{-1}$ ZD2767P was administered intraperitoneally. Groups of 4 mice were killed 1 and 24 h later and the tumours were collected on ice. Using scalpel blades, the tumours were finely chopped at 4˚C and made into a single cell suspension by gentle syringing using a 22-gauge needle. Comet assays were then performed as detailed above.

### Clinical studies

Informed consent both for the trial and for the biopsy procedure was obtained. The protocol was approved by the Cancer Research Campaign Central Institutional Review Board and the Royal Free Hospital Local Ethics Committee (Phase I Trial of Antibody-Directed Enzyme Prodrug Therapy (ADEPT) in Patients with Advanced Colorectal Carcinoma and other CEA-Producing Tumours CRC94/12 Protocol PH1/056). In patients for whom consent was obtained for a tumour biopsy, samples were obtained 1 hour after the last prodrug injection. 10 ml blood for lymphocyte studies was taken at the same time. Liver biopsies were obtained by ultrasound guidance using TruCut technique under local

© 2001 Cancer Research Campaign

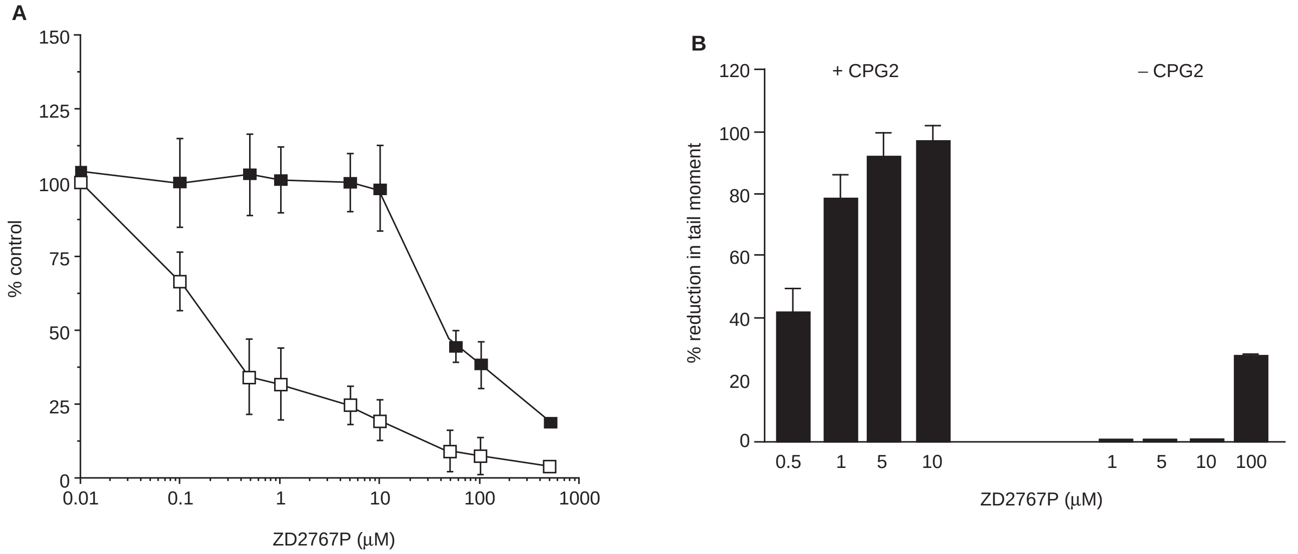

**Figure 1** (**A**) In vitro growth inhibition of ZD2767P and ZD2767P + CPG2 in LS174T colorectal tumour cells. Growth inhibition was assessed in LS174T cells following a 1 h exposure to ZD2767P (■) or ZD2767P + CPG2 (□) using the SRB assay after 4 days in drug free medium. Symbols represent the mean of 4 separate determinations. (**B**) ISCL formation in LS174T colorectal tumour cells following ZD2767P. The cells were treated with 0.5, 1, 10 or 100 µM ZD2767P with or without CPG2 for 1 h before cells were harvested and analysed using the comet assay. Columns represent the mean ± SD from 3 separate experiments anaesthetic. Lymphocytes were separated from whole blood by centrifuging 8 ml of blood in vacutainer CPT™ tubes at 3000 rpm for 20 min. The lymphocytes were washed twice using phosphate buffered saline before analysis. Tumour samples were processed into single cell suspensions as detailed above (in vivo section) and analysed using the comet assay. Localization of the conjugate was achieved by detecting the presence of $I^{131}$ iodine which was attached to the $F(ab')_2$ A5B7 antibody fragment.

### RESULTS

### Growth inhibition and interstrand crosslink production by ZD2767P+/– CPG2 in vitro

The growth inhibitory effect of a 1 h exposure to ZD2767P+/– CPG2 was established for the LS147T human colorectal cell line using the SRB assay (Figure 1a). The concentration of ZD2767P alone resulting in 50% growth inhibition ($IC_{50}$) was 55 ± 9 µM. In contrast, co-incubation of ZD2767P with CPG2 resulted in an $IC_{50}$ of 0.24 ± 0.11 µM. Therefore, ZD2767P was clearly activated by the hydrolytic activity of CPG2.

The critical DNA lesions produced by nitrogen mustards, ISC, can be assessed by a modification of the comet assay as previously described (Hartley et al, 1999). Preliminary investigations with ZD2767 established that the formation of ISC occurs rapidly in cells reaching a peak within 1 hour (data not shown). As seen in Figure 1b, increasing the dose of ZD2767P (+CPG2) resulted in a corresponding increase in ISC following a 1 h exposure to drug. Doses of ZD2767P which resulted in growth inhibition (>10 µM) were also associated with ISC formation. An equitoxic dose of ZD2767P alone and ZD2767P + CPG2 (100 and 0.5 µM respectively) gave a similar level of ISC. There was no detectable DNA strand breakage as assessed by the comet assay at any dose tested (data not shown).

The repair of ISC formed after a 1 hour exposure to 1 µM ZD2767P + CPG2 was measured (Figure 2). In LS147T cells, ISC were repaired rapidly with almost complete removal by 24 hours.

### ISC measurement in LS147T tumour xenografts

Previous work has demonstrated that administration of 75 mg $kg^{-1}$ of ZD2767P 72 h after the conjugate A5B7 + CPG2 resulted in a significant antitumour response in an LS174T xenograft model (18). To determine if the modified comet assay could be used to assess DNA damage in vivo, mice with implanted xenografts of LS174T cells were treated with ZD2767P following administration of the conjugate. The antibody/enzyme conjugate was administered at 25 U/mouse 72 hours prior to injection of prodrug. Two doses of prodrug were used in different groups of mice: therapeutic (75 mg $kg^{-1}$) and subtherapeutic (45 mg $kg^{-1}$) (Blakey et al, 1996). Following sacrifice of mice, biopsies were analysed at 1 and 24 hours following administration of ZD2767P. The results of experiments, using

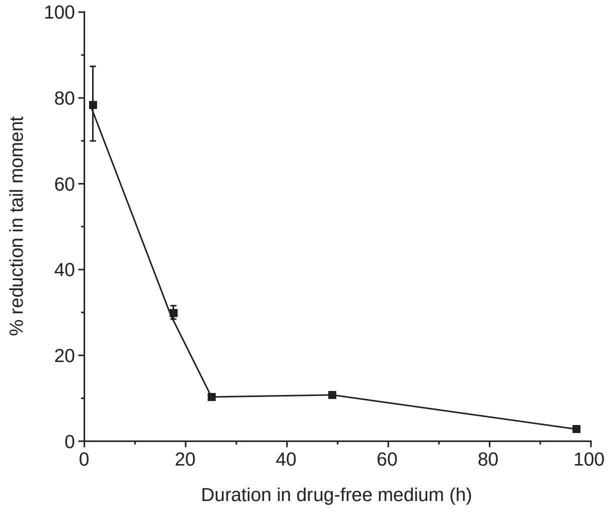

**Figure 2** Formation and repair of ISCL in LS174T colorectal cell line following ZD2767P + CPG2. Cells were treated with 1 µM ZD2767P + CPG2 for 1 h before resuspension in drug-free medium for up to 96 h. At various time points after the initial drug exposure, the cells were harvested and analysed for the presence of ISCL using the comet assay

*© 2001 Cancer Research Campaign* 

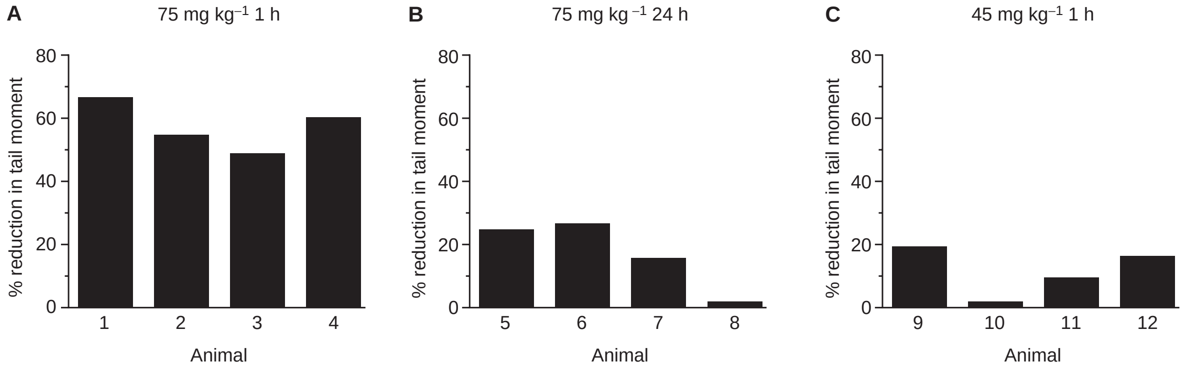

**Figure 3** ZD2767P + CPG2 ISCL formation in vivo. 3 groups of 4 animals were either treated with: (**A**) 75 mg kg$^{-1}$ for 1 h; (**B**) 75 mg kg$^{-1}$ for 24 h or (**C**) 45 mg kg$^{-1}$ for 1 h before tumours were excised and analysed for DNA damage using the comet assay. Untreated animal tumours showed no DNA damage

4 animals for each time point and drug concentration, are indicated in Figure 3. Administration of prodrug (75 mg kg$^{-1}$) for 1 h, 72 h after administration of the antibody/enzyme conjugate, resulted in significant production of ISC (Figure 3A). Biopsies obtained from animals 24 hours after the prodrug administration demonstrated that a significant number of ISC had been repaired as reflected in the < 25% reduction in tail moment (Figure 3B). At 1 hour following administration of 45 mg kg$^{-1}$ there was greatly reduced formation of ISC compared to 75 mg kg$^{-1}$ (Figure 3C). Assays performed on biopsies of tumours from untreated animals showed no evidence of ISC (data not shown). These experiments confirm that the comet assay can be used to measure ISC in vivo, that administration of therapeutic doses is associated with formation of crosslinks, and that the level of detectable lesions is related to the dose of drug administered.

### Analysis of clinical specimens obtained during a clinical study of ADEPT

Having established that the administered dose of ZD2767 significantly correlates with the extent of ISC in vitro and in vivo, we investigated whether there were detectable lesions in patients treated with ADEPT. Samples were obtained during the clinical trial carried out in the Royal Free Hospital; the full results of this Phase I study will be published subsequently. Samples from patients were obtained 1 hour following intravenous injection of ZD2767P. In view of the difficulty of obtaining biopsies within such a limited time from treatment, only 5 specimens could be obtained. These patient samples were analysed as shown in Table 1 with results shown as reduction in tail moment compared with control samples of hepatic metastases from untreated colorectal cancer patients obtained at the time of resection. In addition, bone marrow samples were taken from 2 patients where tumour was not accessible for biopsies. Peripheral blood lymphocytes taken at the same time were also analysed.

**Table 1** Patient clinical data

| Patient No. | Site of metastasis | % reduction in tail moment | |
|---|---|---|---|
| | | Biopsy | Lymphocytes[a] |
| 1 | Liver | 0 | 0 |
| 2 | Liver | 0 | 0 |
| 3 | Liver | 80 | 24 |
| 4 | Liver | 8 | 8 |
| 5 | Liver | 17 | 6 |

[a]From whole blood lymphocytes

There were few detectable ISC in the lymphocytes obtained or in the 2 bone marrow aspirates (data not shown). Tumour biopsy samples also showed no evidence of ISC except in one case (patient 3). In this patient a significant reduction in tail moment was seen following irradiation of the sample. This patient's lymphocytes also showed a lesser (24%) reduction in tail moment indicating some peripheral activation of prodrug. Data from patients 3 and 4 are shown in Figure 4. Following irradiation of biopsies and lymphocytes from the treated patient and an untreated control, a similar increase in tail moment was detected indicating an absence of ISC. In contrast the biopsy from patient 3 showed no alteration in tail moment indicating the presence of ISC. It is of interest that this is the only case of those sampled in which good localization of antibody-enzyme conjugate was observed by phosphorimage analysis and spectrophotometric assay (data not shown). Furthermore this patient demonstrated a response to therapy as shown by decrease in serum CA19–9 levels and stabilization of previously progressive liver metastasis.

### DISCUSSION

The development of novel therapies targeting tumours has extended the scope of phase I clinical trials in assessing feasibility in terms of maximal toxicity. It is increasingly important to directly analyse tumour material to determine if proposed mechanisms of action are operative. The investigation of material obtained following treatment with DNA-damaging agents has been revolutionized by the use of the comet assay. This allows quantitative estimation of DNA damage and repair on small amounts of clinical material following drug exposure, and has been extensively validated. Recent work from our department on lymphocytes from patients receiving ifosfamide therapy demonstrated that modification of this technique allowed measurement of interstrand crosslinks, which are the critical cytotoxic lesions produced by nitrogen mustards. ADEPT has the potential to greatly enhance antitumour selectivity of cancer therapy by activating high concentrations of chemotherapeutic agents selectively at tumour sites. The rationale for ADEPT, previous in vitro studies and clinical experience have been well described (Springer and Niculescu-Duvaz, 1997). In this trial the active chemotherapeutic

 © 2001 Cancer Research Campaign

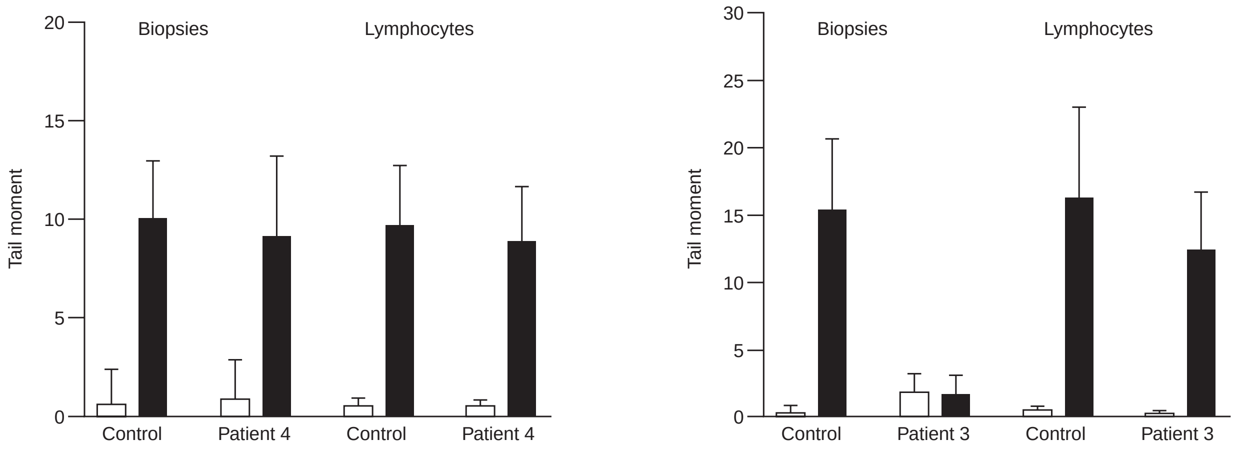

**Figure 4 A and B** DNA tail moments in patient tumour biopsies. After preparation for the comet assay as detailed in the Materials and Methods, irradiated (■) and non-irradiated (□) patient samples were analysed for ISCL. Error bars represent variation within experiment agent is ZD2767, a bisiodophenol mustard with a short half life and high potency which are optimal characteristics for preventing systemic toxicity in a tumour-targeting system. The critical intracellular lesion resulting in cytotoxicity following treatment with bifunctional nitrogen mustards has been demonstrated to be the interstrand crosslink (Sunters et al, 1992). The purpose of this study was to determine the kinetics of crosslink formation and repair in cell culture and xenograft models and to apply this information to the analysis of clinical samples.

Our initial experiments demonstrate that the peak formation of crosslinks occurred within 2 hours. There was a good correlation seen between the extent of crosslinks and cytotoxicity. Repair of lesions was complete by 24 h. There were no detectable single or double DNA strand breaks. The ZD2767P prodrug was active only at >10 μM concentration; at the doses required for cytotoxicity, interstrand crosslinks were detectable. Experiments using the LS174T xenograft model confirmed the ability of the modified comet assay to detect DNA lesions from in vivo studies which had similar time course to cell lines and a similar relationship between dose and crosslink formation.

There are several difficulties in applying these assays to the clinical setting. Apart from the inherent logistical problems in obtaining samples for analysis, the material obtained may not be representative of the tumour as a whole or may be heterogeneous itself. Furthermore there is no possibility of obtaining material before treatment. Based on data obtained from the preclinical studies, we performed biopsies 1 hour following administration of prodrug. However in this study we were only able to obtain tumour samples from 5 patients on the ADEPT study and used material from patients with untreated metastatic cancer or in peripheral lymphocytes from patients who received ADEPT. It is of interest that the one sample in which significant crosslinks could be detected was taken from the patient in whom both good uptake of antibody conjugate and tumour marker response were found. The reduction in tail moment in this sample was in the range found in biopsies from animals given therapeutic doses of ADEPT. This indicates that the degree of ISC in the responding patient corresponded to that found in xenograft models.

In summary this study validates the use of the comet assay in assessing DNA damage in clinical samples with small amounts of tissue. It has demonstrated the feasibility of characterizing and quantitating DNA damage and repair in a variety of clinical settings and will be critical for integrating target validation in further clinical trials of cytotoxic agents.

### ACKNOWLEDGEMENT

In addition to the Cancer Research Campaign we thank AstraZeneca and the Ronald Raven Trust for support.

### REFERENCES

Bagshawe KD, Springer CJ, Searle F, Antoniw P, Sharma SK, Melton RG and Sherwood RF (1998) A cytotoxic agent can be generated selectively at cancer sites. *Br J Cancer* **58**(6): 700–703

Blakey DC, Valcaccia BE, East S, Wright AF, Boyle FT, Springer CJ, Burke PJ, Melton RG and Bagshawe KD (1993) Antitumour effects of an antibody-carboxypeptidas G2 conjugate in combination with a benzoic acid mustard prodrug. *Cell Biophys* **22**: 1–8

Blakey DC, Burke PJ, Davies DH, Dowell RI, East SJ, Eckersley KP, Fitton JE, McDaid J, Melton RG, Niculescu-Duvaz IA, Pinder PE, Sharma SK, Wright AF and Springer CJ (1996) ZD2767, an improved system for antibody-directed enzyme prodrug therapy that results in tumour regressions in colorectal tumour xenografts. *Cancer Res* **56**(14): 3287–3292

Eccles SA, Court WJ, Box GA, Dean CJ, Melton RG and Springer CJ (1994) Regression of established breast carcinoma xenografts with antibody-directed enzyme prodrug therapy against c-erbB2 p185. *Cancer Res* **54**(19): 5171–5177

Fairbairn DW, Olive PL and O'Neill KL (1995) The Comet assay: a comprehensive review. *Mutat Res* **339**: 37–59

Hartley JM, Spanswick VJ, Gander M, Giacomini G, Whelan J, Souhami RL and Hartley JA (1999) Measurement of DNA cross-linking in patients on ifosfamide therapy using the single cell gel electrophoresis (comet) assay. *Clin Cancer Res* **5**(3): 507–512

Melton RG, Boyle JM, Rogers GT, Burke P, Bagshawe KD and Sherwood RF (1993) Optimisation of small-scale coupling of A5B7 monoclonal antibody to carboxypeptidase G2. *J Immunol Methods* **14**: 158(1): 49–56

Napier MP, Sharma SK, Springer CJ, Bagshawe KD, Green AJ, Martin J, Stribbling SM, Cushen N, O'Malley D and Begent RHJ (2000) Antibody-directed Enzme Prodrug Therapy: Efficacy and Mechanism of Action in Colorectal Carcinoma Clin. *Cancer Res* **6**: 765–772

Sherwood RF (1996) Advanced drug delivery reviews: enzyme prodrug therapy. *Advanced Drug Delivery Reviews* **22**: 269–288

Sherwood RF, Melton RG, Alwan SM and Hughes P (1985) Purification and properties of carboxypeptidase G2 from Pseudomonas sp. strain RS-16. Use of a novel triazine dye affinity method. *Eur J Biochem* **148**(3): 447–453

Skehan P, Storeng R, Scudiero D, Monks A, Vistica D, Warren JT, Bokesch H, Kenney S and Boyd MR (1990) New Colorimetric Cytotoxicity Assay for Anticancer-Drug Screening. *J Natl Cancer Inst* **82**: 1107–1112

Spanswick VJ, Hartely JM, Ward TH and Hartely JA (1998) Measurement of drug induced DNA interstrand crosslinking using the single cell gel electrophoresis

© 2001 Cancer Research Campaign 

(Comet) assay In: eds Methods in Molecular Biology: Cytotoxic Drug Resistance Mechanisms. Brown and Brown, Humana Press
Springer CJ and Niculescu-Duvaz I (1997) Antibody-directed enzyme prodrug therapy (ADEPT): a review. *Adv Drug Deliv Rev* **26**: 151–172
Springer CJ, Bagshawe KD, Sharma SK, Searle F, Boden JA, Antoniw P, Burke PJ, Rogers GT, Sherwood RF and Melton RG (1991a) Ablation of human choriocarcinoma xenografts in nude mice by antibody-directed enzyme prodrug therapy (ADEPT) with three novel compounds. *Eur J Cancer* **27**(11): 1361–1366
Springer CJ, Antoniw P, Bagshawe KD and Wilman DE (1991b) Comparison of the half-lives and cytotoxicity of N-chloroethyl-4-amino and N-mesyloxyethyl-benzoyl compounds, products of prodrugs in antibody-directed enzymes prodrug therapy (ADEPT). *Anti-Cancer Drugs Design* **6**: 467–479
Springer CJ, Dowell R, Burke PJ, Hadley E, Davis DH, Blakey DC, Melton RG and Niculescu-Duvaz I (1995) Optimisation of alkylating agent prodrugs derived from phenol and aniline mustards: a new clinical candidate prodrug (ZD2767) for ADEPT. *J Med Chem* **38**: 5051–5065
Sunters A, Springer CJ, Bagshawe KD, Souhami RL and Hartley JA (1992) The cytotoxicity, DNA crosslinking ability and DNA sequence selectivity of the aniline mustards melphalan, chlorambucil and 4-[bis(2-chloroethyl)amino] benzoic acid. *Biochem Pharmacol* **44**: 59–64

*© 2001 Cancer Research Campaign*
